# Supplementary material for: MetaQTL: a package of new computational methods for the meta-analysis of QTL mapping experiments
Source: BMC Bioinformatics. 2007 Feb 8;8:49. doi: 10.1186/1471-2105-8-49 (PMC1808479; doi:10.1186/1471-2105-8-49)
Supplement: Additional File 7 — MetaQTL Package : jar file and tutorial. This Zip archive contains both the MetaQTL JAR file and the files of the tutorial. [file 1471-2105-8-49-S7.zip › org.inra.metaqtl/doc/org/metaqtl/bio/IBioLocus.html]

IBioLocus


|  |  |  |
| --- | --- | --- |
| **Overview** \| **Package** \| **Class** \| **Use** \| **Tree** \| **Deprecated** \| **Index** \| **Help** | |  |
| **PREV CLASS**   **NEXT CLASS** | **FRAMES**    **NO FRAMES**     **All Classes** |  |
| SUMMARY: NESTED \| FIELD \| CONSTR \| METHOD | DETAIL: FIELD \| CONSTR \| METHOD |  |


---


## org.metaqtl.bio Interface IBioLocus

**All Superinterfaces:**: IBioAdaptable, IBioEntity

**All Known Implementing Classes:**: AlleleContainer, Locus, Marker, QTL

---

``` public interface IBioLocus extends IBioEntity ```

**Author:**
:   Jean-Baptiste Veyrieras

---

| **Field Summary** | |
| --- | --- |
| `static int` | `MARKER` |
| `static int` | `QTL` |
| `static java.lang.String[]` | `TypesString` |


| **Method Summary** | |
| --- | --- |
| `void` | `addAllele(IBioAllele allele)` |
| `IBioAllele[]` | `alleles()` |
| `IBioAllele` | `getAllele(java.lang.String name)` |
| `int` | `getAlleleNumber()` |
| `IBioLGroup` | `getGroup()` |
| `int` | `getLocusType()` |
| `ILGroupPosition` | `getPosition()` |
| `int` | `getType()`             There are 2 main class of entities. |
| `void` | `removeAllele(java.lang.String name)` |
| `void` | `setGroup(IBioLGroup group)` |
| `void` | `setPosition(double position)` |
| `void` | `setPosition(double position, double sd)` |
| `void` | `setPosition(ILGroupPosition position)` |

**Methods inherited from interface org.metaqtl.bio.IBioEntity**

`getName, getParent, getProperties, setName, setProperties`

**Methods inherited from interface org.metaqtl.bio.IBioAdaptable**

`getBioAdapter`

**Field Detail**

### MARKER

```
static final int MARKER
```

**See Also:**: Constant Field Values

---


### QTL

```
static final int QTL
```

**See Also:**: Constant Field Values

---


### TypesString

```
static final java.lang.String[] TypesString
```


**Method Detail**

### alleles

```
IBioAllele[] alleles()
```

---


### addAllele

```
void addAllele(IBioAllele allele)
```

---


### removeAllele

```
void removeAllele(java.lang.String name)
```

---


### getAllele

```
IBioAllele getAllele(java.lang.String name)
```

---


### getGroup

```
IBioLGroup getGroup()
```

---


### setGroup

```
void setGroup(IBioLGroup group)
```

---


### getAlleleNumber

```
int getAlleleNumber()
```

---


### getPosition

```
ILGroupPosition getPosition()
```

---


### setPosition

```
void setPosition(ILGroupPosition position)
```

---


### setPosition

```
void setPosition(double position)
```

---


### setPosition

```
void setPosition(double position,
                 double sd)
```

---


### getType

```
int getType()
```

:   **Description copied from interface: `IBioEntity`**
:   There are 2 main class of entities. The first one deals with population
    biological entity, i.e population itself and individuals. The second class
    is a representation of microscopic biological entity from genome container
    to alleles.

    :   **Specified by:**: `getType` in interface `IBioEntity`

---


### getLocusType

```
int getLocusType()
```


---


|  |  |  |
| --- | --- | --- |
| **Overview** \| **Package** \| **Class** \| **Use** \| **Tree** \| **Deprecated** \| **Index** \| **Help** | |  |
| **PREV CLASS**   **NEXT CLASS** | **FRAMES**    **NO FRAMES**     **All Classes** |  |
| SUMMARY: NESTED \| FIELD \| CONSTR \| METHOD | DETAIL: FIELD \| CONSTR \| METHOD |  |


---
